# Application of Perinatal Derivatives on Oncological Preclinical Models: A Review of Animal Studies

**DOI:** 10.3390/ijms23158570

**Published:** 2022-08-02

**Authors:** Ricardo Teixo, Ana Salomé Pires, Eurico Pereira, Beatriz Serambeque, Inês Alexandra Marques, Mafalda Laranjo, Slavko Mojsilović, Roberto Gramignoli, Peter Ponsaerts, Andreina Schoeberlein, Maria Filomena Botelho

**Affiliations:** 1 Coimbra Institute for Clinical and Biomedical Research (iCBR) Area of Environment, Genetics and Oncobiology (CIMAGO), Institute of Biophysics, Faculty of Medicine, University of Coimbra, 3000-548 Coimbra, Portugal; ricardo.teixo@gmail.com (R.T.); euricojgp@gmail.com (E.P.); beatrizprazserambeque@gmail.com (B.S.); ines.marques@student.uc.pt (I.A.M.); mafaldalaranjo@gmail.com (M.L.); mfbotelho@fmed.uc.pt (M.F.B.); 2 Center for Innovative Biomedicine and Biotechnology (CIBB), University of Coimbra, 3000-548 Coimbra, Portugal; 3 Clinical Academic Center of Coimbra (CACC), 3000-548 Coimbra, Portugal; 4 Faculty of Pharmacy, University of Coimbra, 3000-548 Coimbra, Portugal; 5 Group for Hematology and Stem Cells, Institute for Medical Research, University of Belgrade, 11129 Belgrade, Serbia; slavko@imi.bg.ac.rs; 6 Division of Pathology, Department of Laboratory Medicine, Karolinska Institutet, 171 77 Stockholm, Sweden; roberto.gramignoli@ki.se; 7 Department of Pathology, Medicinsk Cancer Diagnostik, Karolinska University Hospital, 171 64 Huddinge, Sweden; 8 Laboratory of Experimental Hematology, Vaccine and Infectious Disease Institute (Vaxinfectio), University of Antwerp, 2610 Antwerp, Belgium; peter.ponsaerts@uantwerpen.be; 9 Department of Obstetrics and Feto-Maternal Medicine, Inselspital, Bern University Hospital, University of Bern, 3010 Bern, Switzerland; andreina.schoeberlein@dbmr.unibe.ch; 10 Department for BioMedical Research (DBMR), University of Bern, 3012 Bern, Switzerland

**Keywords:** perinatal derivatives, cancer, preclinical studies, animal models

## Abstract

The increasing cancer incidence has certified oncological management as one of the most critical challenges for the coming decades. New anticancer strategies are still needed, despite the significant advances brought to the forefront in the last decades. The most recent, promising therapeutic approaches have benefitted from the application of human perinatal derivatives (PnD), biological mediators with proven benefits in several fields beyond oncology. To elucidate preclinical results and clinic outcomes achieved in the oncological field, we present a narrative review of the studies resorting to animal models to assess specific outcomes of PnD products. Recent preclinical evidence points to promising anticancer effects offered by PnD mediators isolated from the placenta, amniotic membrane, amniotic fluid, and umbilical cord. Described effects include tumorigenesis prevention, uncontrolled growth or regrowth inhibition, tumor homing ability, and adequate cell-based delivery capacity. Furthermore, PnD treatments have been described as supportive of chemotherapy and radiological therapies, particularly when resistance has been reported. However, opposite effects of PnD products have also been observed, offering support and trophic effect to malignant cells. Such paradoxical and dichotomous roles need to be intensively investigated. Current hypotheses identify as explanatory some critical factors, such as the type of the PnD biological products used or the manufacturing procedure to prepare the tissue/cellular treatment, the experimental design (including human-relevant animal models), and intrinsic pathophysiological characteristics. The effective and safe translation of PnD treatments to clinical practice relies on the collaborative efforts of all researchers working with human-relevant oncological preclinical models. However, it requires proper guidelines and consensus compiled by experts and health workers who accurately describe the methodology of tissue collection, PnD isolation, manufacturing, preservation, and delivery to the final user.

## 1. Introduction

Cancer is the second leading cause of death worldwide, and its present ranks indicate that, during this century, it may overcome cardiovascular diseases as the leading cause of premature death in most countries. Indeed, cancer has already overcome cardiovascular diseases in countries with a higher Human Development Index (HDI) [1]. Recent estimations offered by Globocan 2020 report an incidence of 19.29 million cases and 9.96 million deaths worldwide in 2020, resulting in one death in six patients for oncological reasons [2,3]. Dramatically, a 47% increase in incidence rates is expected by 2040 [3].

The increasing cancer incidence makes cancer management a major challenge for the following decades. As one of the main pillars of the cancer control spectrum, cancer management must ensure the availability of highly effective therapeutic options and equal access to those therapies. It has been highlighted that the poorest outcomes in cancer control characterize developing countries as a direct consequence of social and economic inequalities in cancer management [2]. So, it is imperative to create new and affordable therapeutic strategies.

Current treatment options include surgery, hormone therapy, chemotherapy, targeted therapy, radiation therapy, immunotherapy, or its combination [4]. Surgery is usually the preferred upfront treatment; however, several tumors are not eligible for surgical resection. Chemotherapy, as systemic therapy, inevitably induces high toxicity levels in healthy surrounding tissues [4]. The identification of several oncogene drivers led to the development of safer and more effective targeted therapies. However, reported resistance, drug toxicity, and high costs hamper expanded use and limit patients’ access to any therapeutic approach [5]. Although immunotherapy proved powerful clinical outcomes based on activating the immune system to fight cancer, adverse effects of autoimmunity and non-specific inflammation are a concern. Additionally, high costs associated with the preparation of autologous cellular products still limit such cellular approaches on a large scale [6].

Another recent promising anticancer strategy includes the application of human perinatal derivatives (PnD). PnD biological products include perinatal tissues and derivatives as cells or their secretome. PnDs have proven clinical or biological benefits in several fields beyond oncology, like wound-healing [7,8,9,10], COVID-19 [11,12], ovarian diseases [13], neuroprotection [11,14], bone regeneration [15], cardiovascular repair and regeneration [16], ophthalmology [17,18], and more.

The attractiveness of PnDs for therapeutic purposes is justifiable by reduced ethical concerns, since the human term placenta is considered a biological waste, there are reduced costs and ease of collection, processing, and handling of this “raw material”. Additionally, it has been largely described that PnD products present low or absent expression of human leukocyte antigens and co-stimulatory molecules, supporting allogeneic administration/transplantation [19]. The reported activities offered by some PnDs have advocated their anticancer therapeutic effect [20]. However, recent results unraveled a dual role in tumor cell growth, challenging PnD use and therapeutic potential in the oncology field [19].

In the last decades, several studies have demonstrated that stem cells could possess intrinsic antitumor effects and intrinsic tropism toward malignant cells [21]. The human PnD have become a valuable source of mesenchymal stromal/stem cells (MSC) [22]. MSC have been described as possessing several important features critical for oncological treatments: low immunogenicity and immunomodulatory effects, anti-inflammatory properties, no tumorigenicity, low risk of viral infection and no ethical considerations for their use, the capacity for pre-loading with bioactive cargo that, together with the capacity of homing in different tissues, emerged as promising vehicles for gene and cellular therapy [23,24,25,26,27,28,29]. MSC have shown high proliferation and differentiation ability, migration capacity toward tumors, and growth inhibition of certain tumors [28,29]. MSC may inhibit tumor growth by targeting Akt activity, inhibiting angiogenesis, promoting apoptosis, and activating antitumor pathways [30]. Conversely, MSC can contribute to tumor progression by secretion of tumor-supportive immunosuppressive factors, inhibition of antitumor immune response, and promotion of angiogenesis by increasing pro-angiogenic factors, such as MIP-2, VEGF, TGF-β, and IL-6, which shows their very controversial role in this research field [30].

To understand where we stand in the therapeutic effects of PnDs, a team composed of pioneers and experts in using PnD bioproducts in oncological treatments, afferent to the COST SPRINT Action, compiled a narrative review based on a systematic literature search. The primary goal was to report the animal models instrumental in assessing the benefit of PnD therapy. We also describe different studies regarding the type of PnD used, interventions performed, disease targets, route and dose of administration, time of exposure, and outcomes.

## 2. Search Strategy and Data Collection Methodology

The literature search and data collection were done in the scope of the work developed by the scientific network of the COST SPRINT Action (CA17116) in a coordinated effort to improve the understanding of the benefits of PnD therapy based on the available in vivo experimental models. The search strategy applied, the selection of studies and inclusion criteria, and the data management and extraction methodology were previously described in detail [31]. The articles identified in the oncology field account for 73 contributions, covering different PnD products: human placenta cells (11), amniotic fluid cells (5), amniotic membrane derivatives (9), decidua (2), and human umbilical cord derivatives (46).

**Figure 1:**
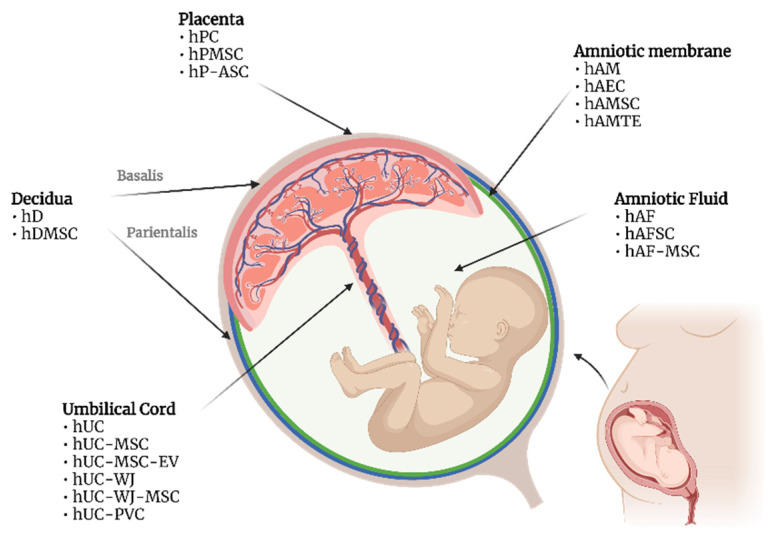
PnD used in oncological animal models. Nomenclatures follow the Consensus for Tissue and Cell Nomenclature recently published by the COST SPRINT Action (CA17116) consortium: MSC—mesenchymal stromal/stem cells; hPC—human placenta cells; hPMSC—human placenta-derived mesenchymal stromal/stem cells; hP-ASC—human placenta-derived adherent stromal cells; hAM—human amniotic membrane; hAEC—human amniotic membrane epithelial cells; hAMSC—human amniotic membrane MSC; hAMTE—human amniotic membrane tissue extract; hD—human decidua; hDMSC—human decidua MSC; hAF—human amniotic fluid; hAFSC—human amniotic fluid stromal/stem cells; hAF-MSC—human amniotic fluid MSC; hUC—human umbilical cord; hUC-MSC—human umbilical cord MSC; hUC-MSC-EV—hUC-MSC-derived extracellular vesicles; hUC-WJ-MSC—human umbilical cord Wharton’s jelly MSC; hUC-PVC—human umbilical cord perivascular cells.

Considering the high variability reported in the literature on terminologies and abbreviations adopted by different authors, we harmonized the terms according to the Consensus for Tissue and Cell Nomenclature recently published by the COST SPRINT Action (CA17116) consortium (Figure 1) [27]. Briefly, the abbreviations adopted include human placenta-derived mesenchymal stromal/stem cells (hPMSC), human placenta-derived adherent stromal cells (hP-ASC), human amniotic fluid stromal/stem cells (hAFSC), human amniotic fluid MSC (hAF-MSC), human amniotic membrane epithelial cells (hAEC), human amniotic membrane MSC (hAMSC), human amniotic membrane tissue extract (hAMTE), human decidua MSC (hDMSC), human umbilical cord MSC (hUC-MSC), human umbilical cord Wharton’s jelly MSC (hUC-WJ-MSC), human umbilical cord perivascular cells (hUC-PVC), and hUC-MSC-derived extracellular vesicles (hUC-MSC-EV).

## 3. Placenta Cells

According to the Consensus on Perinatal Derivatives, human placenta cells (hPC) refer to a heterogenous collection of cell types obtained from the placenta [27]. The role of hPC has been investigated in the prevention of tumorigenesis and in tumor development, but mainly as an anticancer therapeutic approach in several types of neoplasia.

A strategy to prevent carcinogenesis using hPMSC was described in gastric cancer. A model of *Helicobacter pylori*-infected disease was obtained by inoculation in C57BL/6 mice. hPMSC, as well as their conditioned medium, were used as a therapeutic approach to atrophic gastritis. Treated animals showed that hPMSC and the conditioned medium significantly reduced inflammation and gastric atrophy, contributing to a reversion to a normal environment and preventing the development of gastric precancerous lesions triggered by *Helicobacter pylori* [32].

To explore the role of hPMSC in in vivo colon cancer models, male BALB/c nude mice were inoculated with HCT116-GFP cells, and when tumors reached 50 mm3, hPMSC were repeatedly administered through the tail vein. Animals subjected to hPMSC administration showed significantly superior tumor volume to the control group. Moreover, these cells induced a colon cancer stem cells (CSC) phenotype, contributing to increased CD133 expression and colon cancer cells spread [33].

Extensive investigations on hPMSC have focused on their promise as an anticancer therapeutic approach, largely due to their capacity for homing to tumors [33]. It becomes essential to validate methods that allow exploring these characteristics in animal models. A xenograft model of HepG2 hepatocellular carcinoma in male C57BL/6 nude mice showed a superior homing capacity of hPMSC administrated intratumorally than intravenously [34]. To validate the efficacy of hPMSC as a cell-based vector for targeting glioblastoma CSC, donor allocation was tracked in real-time through fluorescence imaging and magnetic resonance imaging (MRI). Normal mice and orthotopic glioblastoma models were intravenously or intraperitoneally inoculated with hPMSC. The initial accumulation of donor cells hPMSC in the lung was rapidly resolved within one week. The tropism of hPMSC to glioblastomas was confirmed by MRI studies [35].

The antitumor action of engineered hPMSC has also been studied as a potential gene delivery vector for targeted therapy in several human malignancies. For example, hPMSC as carriers of adenoviruses expressing pigment epithelium-derived factor (PEDF) were evaluated regarding their therapeutic potential against melanoma. In this study, hPMSC were isolated and characterized, and afterward, these MSC were engineered through adenoviral transduction, originating hPMSC-PEDF. Melanoma xenografts were subcutaneously induced in female C57BL/6 mice and submitted to treatment with hPMSC or modified hPMSC. Animals treated with hPMSC-PEDF presented a significant decrease in the tumor volume and expressive cell apoptosis compared with animals treated with hPMSC or PBS. No differences were observed between the last beforementioned groups [23]. Two additional studies explored the anticancer effect of hPMSC expressing endostatin (Endo), obtained through adenoviral transduction, as a gene delivery therapy for ovarian cancer and colorectal peritoneal carcinomatosis (CRPC) [36,37]. Female BALB/c mice received the A2780s ovarian cancer or the CT26 CRPC cells intraperitoneally. Subsequently, hPMSC or ndo-hPMSC were administered intraperitoneally. Both studies revealed that Endo-hPMSC promoted the inhibition of the tumor’s development, angiogenesis, and cell proliferation, inducing apoptosis [36,37]. Moreover, Endo-hPMSC showed the ability for ovarian tumor homing [36]. More recently, the effectiveness of engineered hPMSC as a vector of therapeutic genes was investigated as a treatment modality for colon cancer. In this study, transduced hPMSC, hPMSC-DF (herpes simplex virus truncated thymidine kinase and firefly luciferase), and hPMSC-DF + GCV (hPMSC-DF combined with ganciclovir) were administered in male Nu/Nu nude mice. The action of hPMSC-DF on the colon tumor’s xenografts growth, proliferation, and its potential for anticancer effect was evaluated. hPMSC-DF induced partial tumor growth inhibition and showed the ability to migrate to the tumor area.

Furthermore, hPMSC-DF also inhibited tumor proliferation, which is more prominent in the animal group receiving hPMSC-DF + GCV. This inhibitory effect was also due to the apoptosis of colon tumors mediated by hP-MSC-DF and hPMSC-DF + GCV [24]. These studies demonstrate the potential of hPMSC as gene delivery vectors for targeted anticancer therapy, regardless of the type of cancer.

The combination of hPMSC with other therapeutic approaches is an alternative strategy explored to eradicate human malignant diseases. A study evaluated the combination of sorafenib and hPMSC as a treatment modality for hepatocellular carcinoma. Male BALB/c nude mice were inoculated with 1 × 10^7^ HepG2 cells to establish a heterotopic model of hepatocellular carcinoma. When tumors presented a volume superior to 100 mm^3^, animals were submitted to treatment with sorafenib (intraperitoneal), hPMSC (intratumoral), or both. Findings demonstrated that this combination induced a prominent inhibitory effect of tumor spreading, even as tumor cell apoptosis, enhancing the sorafenib action. No effect was observed for hPMSC per se on tumor proliferation [38]. In another study, hPMSC were modified to carry the tumor necrosis factor-related apoptosis-inducing ligand (TRAIL-hPMSC) and combined these engineered hPMSC with curcumin-loaded chitosan nanoparticles (Cu–NPs) targeting a model of triple-negative breast cancer. A heterotopic model of breast cancer was established in female BALB/c mice through a subcutaneous inoculation of 4T1 cells. When tumors become palpable, hPMSC, Cu–NPs, and TRAIL-hPMSC, or TRAIL-hPMSC and Cu–NPs, were injected into the tumor site. This combined therapeutic approach inhibited tumor proliferation and promoted apoptosis of triple-negative breast cancer in vivo. However, hPMSC and Cu–NPs did not reveal a significant antitumor effect [39]. Hereupon, combined therapeutic modalities seem to achieve effective antitumor outcomes, despite the monotherapy with hPMSC showing modest results in different tumors.

Another type of hPC, human placental-derived adherent stromal (hP-ASC), was tested regarding their potential antitumor effect. hP-ASC were submitted to a co-induction with the tumor necrosis factor alpha (TNF-α) and interferon gamma (IFN-γ). Two different triple-negative breast cancer models were established. First, a heterotopic model was developed through a subcutaneous inoculation of MDA-MDB-231 cells in the animals’ backs. Induced and non-induced hP-ASC were intramuscularly administered to these animals. The second orthotopic model was obtained by inoculating MDA-MDB-231 cells directly in the mammary fat pad. In the induced hP-ASC group, PlasmaLyte (an isotonic electrolyte solution) was administered weekly from day 6 to 41, followed by induced hP-ASC administration from day 48 to 83. After animals’ occision, histological analysis was performed. Tumor development was notably different between animal model groups treated with induced hP-ASC, which presented more efficacy in orthotopic tumors as tumor progression was delayed. Complete response was achieved in 30% of animals treated with induced hP-ASC [40].

## 4. Amniotic Fluid Cells

Human amniotic fluid stromal/stem cells (hAFSC) and human amniotic fluid mesenchymal stromal/stem cells (hAF-MSC) obtained from amniotic fluid samples from patients undergoing amniocentesis have been investigated.

The expression of Oct4 in hAFSC advocates for an intermediate pluripotent-multipotent stage between human embryonic stem cells and lineage-restricted adult stem cells, without chromosomal abnormalities or risk in teratomas formation [41,42]. These cells present tumor-tracking properties, providing an attractive cell therapy vehicle [42].

Also, hAFSC were successfully used as gene therapy carriers in subcutaneous xenograft animal models of lung cancer. DAL-1 was evaluated as a candidate for lung cancer gene therapy since its expression is lost in various lung cancers, and overexpression significantly suppressed the proliferation and invasion and promoted cell apoptosis. Further, hAFSC were constructed to overexpress the CXCR4 promoter-driven DAL-1. Tumor xenografts were established subcutaneously in BALB/c nude mice by inoculating 2 × 10^6^ A549 human lung cancer cells. Constructed hAFSC were injected intratumorally or in the tail vein, after which hAFSC homing and virus replication was observed via tail vein. Both approaches led to reduced growth and more significant necrosis than null vector-treated animals, pointing to hAFSC as promising gene therapy vehicles targeting lung cancer and decreasing tumorigenesis [41].

In another cell therapy strategy, hAFSC were used to deliver suicide genes into the site of tumor formation. Suicide genes encode for viral or bacterial enzymes that can convert non-toxic prodrugs into toxic metabolites that induce tumor cell death. Cytosine deaminase converts 5-fluorocytosine into 5-FU, and herpes simplex virus thymidine kinase catalyzes ganciclovir, leading to a toxic form [42]; here, hAFSC were used as vehicles of these two suicide genes for breast cancer. Cell therapy was performed on female BALB/c nude mice previously inoculated with MDA-MB-231 cells into the mammary fat pads. Then the hAFSC were injected circumtumorally, and intraperitoneal injections of 5-fluorocytosine and ganciclovir completed the treatment. Despite cell therapy groups showing a tumor volume equivalent to animals submitted to 5-FU only, the first conserved breast tissue structures versus destruction on 5-FU-treated mice [42].

Notably, hAF-MSC, like any other MSC, are particularly attractive cellular mediators due to their manipulation advantages (easily isolated and expanded in culture, low immunogenicity). They have been successfully genetically manipulated using current molecular techniques [43], serving as vehicles [44,45,46]. Still, safety is an issue based on contradictory reports where tumor growth arrest and promotion arose [44].

In another case, hAF-MSC were investigated regarding ovarian cancer tropism and the ability for targeted therapy [46,47]. Nude mice were subcutaneously inoculated with the hAF-MSC or SKOV3 cells in the right scapular. Tumors were observed only on SKOV3 inoculated mice, pointing to the safe use of hAF-MSC [46]. Another group of mice inoculated with SKOV3 cells was submitted to hAF-MSC injection in the tail vein. Cells were primarily found in the tumor site, and a small number appeared in the liver and spleen [46]. In another study, SKOV3 ovarian cancer cells were inoculated subcutaneously in the scapula region of the Balc/c nude mice. When tumors reached one centimeter in diameter, the animals were intravenously injected with hAF-MSC stably transfected to express IL-2. Cells presented high motility during migration in vivo, reaching the tumor site and secreting IL-2 locally [47]. These studies showed the absence of tumorigenicity and high motility to migrate to ovarian cancer sites, emphasizing the potential of hAF-MSC to be used as therapy vehicles [46,47].

In one study, hAF-MSC migratory potential and tumor homing ability were evaluated in a bladder cancer animal model obtained by cell (T24M cells) inoculation on NOD-SCID mice tail base. hAF-MSC were injected into the lateral tail vein. Cells were found infiltrated into the tumor and at its periphery, but a few were also found in the lung. Additionally, INF-β-carrying cells significantly retarded tumor growth [45]. In fact, type I interferon would be an exciting option for cervical cancer if cytotoxicity could be obviated. Also, hAF-MSC were used to deliver IFNα to cervical tumor sites of BALB/c nude mice subcutaneously inoculated with HeLa cells. The cells selectively migrated to the tumor sites, participated in tumor construction, and promoted tumor growth. However, the genetically modified to overexpress IFNα counterparts suppressed angiogenesis and arrested tumor growth [48].

In another case, hAF-MSC were also as the delivery vehicle for endostatin, a potent anti-angiogenic and a secretable form of carboxylesterase 2, able to convert the prodrug CPT11 into active SN-38, a drug associated with high toxicity. Glioblastoma animal models were obtained in BALB/c nude mice stereotactically inoculated in the left striata. A potent antitumor effect was achieved with low CPT11 doses and decreased endothelial and proliferation markers [43]. In a model of recurrence after surgical resection (subcutaneous injection plus surgery to remove 90% of the mass), hAF-MSC were implanted in the surgical cavity along with CPT11 treatment. The treatment showed clear suppression of tumor regrowth [43].

Recent in vivo studies in immunocompetent (BALB/c) and immunocompromised (NOD-SCID) mice explored the effects of IFN-β and IFN-γ priming. H460 lung carcinoma cells were implanted after being admixed with primed hAF-MSC; hAF-MSC or IFN-γ-primed hAF-MSC promoted the earlier onset and delayed xenograft disappearance. The opposite result was observed with IFN-β- or IFN-β and IFN-γ-primed hAF-MSC [44].

## 5. Amniotic Membrane Derivatives

The amniotic membrane is the innermost placental layer around the amniotic fluid and the fetus during pregnancy. Amnion membranes, as well as amniotic fluid, are fetal-derived tissues. Both epithelial and mesenchymal cells have been investigated regarding their anticancer properties. The epithelial cells paving the fetal side of the amnion membrane have been characterized by many properties previously described on MSC [49,50]. The ability of human amniotic membrane epithelial cells (hAEC) to stimulate antitumor responses was investigated in preventive and treatment approaches. Vaccination of BALB/c mice with both hAEC and gamma-irradiated CT26 cells prevented the development of colon adenocarcinoma tumors in a subsequent challenge. Vaccinated mice mounted tumor-specific Th1 responses and produced cross-reactive antibodies against cell surface markers of cancer cells. Other groups of BALB/c and C57BL/6 vaccinated mice were challenged with 4T1 breast cancer and B16F10 melanoma cells, respectively. While vaccination did not protect against breast cancer, inhibition of melanoma development was complete [50]. In another approach, hAEC were co-injected with SK-OV-3 cells into BALB/c nude mice. Co-injection tumors presented significantly lower average weight and volume than xenografts obtained from cancer cells, only [49].

The use of hAEC as cell therapy vectors was explored in breast cancer models. Female BALB/c nude mice were inoculated in the right mammary fat pad with MDA-MB-231 breast cancer cells. Seven weeks after tumor implantation, mice were treated with circumtumoral injections of PBS, 5-FU, or hAEC. Cell therapy led to significantly decreased tumor volumes and the absence of side effects compared to the 5-FU-treated mice. Moreover, breast tissues appeared intact following cell therapy, contrasting with almost complete destruction in the 5-FU group [51].

Very little is known about the epithelial cells isolated from rat amniotic membrane. Male C57BL/6J mice were subcutaneously inoculated with B16F10 melanoma cells, amniotic epithelial cells, and a mixture of these two cell types in different proportions. Despite a significant delay in tumor growth, this finding was not corroborated by tumor weight [52].

Human amniotic membrane MSC (hAMSC) were investigated as another possibility for glioma cell therapy. Male BALB/c nude were subcutaneously implanted with C6 cells, followed by intratumoral injections of HUVECs. The administration of hAMSC significantly reduced tumor size. Moreover, xenografts analysis showed evidence of apoptosis, namely increased Bax, caspase-8, and caspase-3 and diminished Bcl-2 expression. This study demonstrated the potential to use these cells as therapy vehicles for gliomas [53].

In a pioneering approach, Mamede et al. used a full human amniotic membrane tissue extract (hAMTE) to treat oncological disease. Xenografts of hepatocarcinoma HepG2 and HUH7 cells were obtained in BALB/c mice, and the extract was administered intraperitoneally every 48 h for 12 days; however, tumor regression was observed only for HepG2-derived tumors [54].

## 6. Decidua

Human decidua (hD) is a component of the placenta with maternal origin derived from the endometrium. According to the consensus on PnD, there are three types of hD layers distinguished based on the spatial relation to the implanting embryo: human basal decidua, human capsular decidua, and human parietal decidua [27].

The potential of hDMSC as an anticancer agent has been sought in rat N-nitroso-N-methylurea (NMU)-induced mammary tumors, a model of mammary carcinogenesis similar to hormone-dependent breast cancer. The rat model was developed by a weekly administration of 5 mg per 100 g of body weight of NMU for two weeks, plus the addition of metoclopramide (0.125 mg/L) to drinking water to increase and accelerate the induction of tumors. As soon as primary tumors were detected, rat NMU-induced mammary tumors were weekly injected with 1.5 × 10^6^ hDMSC, previously isolated by enzymatic digestion from the hD of placental membranes of healthy mothers, into the rat tail vein, for five weeks. The in vivo migration of administered cells into circulation was analyzed after 24 h and 72 h in mammary tumors, liver, and lungs. A specific tropism and homing displayed by injected hDMSC was observed by Vegh and colleagues in the NMU-induced mammary tumors. Moreover, hDMSC seems to play an important role in NMU primary tumor evolution and progression by decreasing tumor size and growth after injection and delaying and inhibiting secondary tumors’ development in hDMSC-treated rats compared to vehicle-treated rats [29].

Paris and colleagues [28] exploited the potential of hDMSC as a cellular vehicle for carrying drug-loaded NPs to induce breast cancer cell death. To do so, hDMSC were isolated by enzymatic digestion of hD and later loaded with fluorescent NPs. The NPs were fast taken up by cells in around two hours and stayed retained in the cells for an extended period, up to 5 days. For in vivo analysis, 10^6^ NPs loaded hDMSC were injected into the tail vein of Sprague–Dawley rats with NMU mammary tumors. The analysis of the tumors three days post-injection revealed the presence of NPs around some tissue nuclei inside the tumors, indicating that the homing capacity of hDMSC towards tumors is not changed when carrying NPs. The potential of using hDMSC for carrying NPs loaded with anticancer drugs as chemotherapeutics is still to be addressed in vivo [28]. Together, these features highlight the capacity of hDMSC as effective transport vehicles for NPs to target disease site and their potential as a platform for anticancer therapy.

## 7. Umbilical Cord Derivatives

The COST SPRINT Action (CA17116) consensus highlights the need for standardization on the protocols for isolating MSC from the human umbilical cord (hUC) [27]. However, studies published so far do not gather consensus and do not always specify from which region of hUC the cells were isolated. In cases where no specific details are provided on the regions of hUC from which cells are extracted, they will be referred to as human umbilical cord mesenchymal stromal/stem cells (hUC-MSC).

### 7.1. Human Umbilical Cord Mesenchymal Stromal/Stem Cells (hUC-MSC)

The use of hUC-MSC in cancer animal models has been widely reported. For example, hUC-MSC tumorigenicity has been studied in a mouse model, where intravenous injection of hUC-MSC did not induce tumors in the animals. On the other hand, injection of mouse embryonic stem cells ES-3 leads to tumor formation in the lungs, severe dyspnea symptoms, and minimal activity only six weeks after injection. Overall results suggest that hUC-MSC could be a promising and safe treatment for clinical uses [55].

The targeting of tumor cells by hUC-MSC has been exploited by intratumoral or contralateral ventricular administration in a murine glioma model. After contralateral injection, the hUC-MSC migrated to the glioma cells through the corpus callosum, while intratumoral injection led the cells to accumulate between the tumor and the tumor bed [56].

Tumor tropism of hUC-MSC towards breast tumor cells has been assessed by developing an animal model with cells expressing reporter genes for bioluminescence imaging. Injection of hUC-MSC led to decreased tumor growth compared to control group [57]. Moreover, a significant decrease in angiogenesis and a significant increase in tumor cell apoptosis induced by hUC-MSC were demonstrated [57].

In the case of multiple myeloma, it was demonstrated that hUC-MSC suppresses multiple myeloma growth in vivo. The NOD.CB17-Prkdcscid/J mice developed the multiple myeloma animal model by subcutaneous administration of 1 × 10^6^ RPMI-8226 cells in the abdomen. The hUC-MSC were administered (2 × 10^6^) simultaneously with the tumor cells or seven days later through peritumoral injection. The simultaneous injection of hUC-MSC inhibited the tumor growth by 50% compared to controls. In contrast, the peritumoral inoculations of hUC-MSC decreased the tumor size (twenty times) compared to controls, 30 days post-implantation [58].

To evaluate the tumor formation, the hUC-MSC were administered subcutaneously at the flank region (2 × 10^6^ or 1 × 10^7^) or intravenously via the lateral tail vein (2 × 10^6^ or 3 × 10^6^ or 6.5 × 10^6^) in CB17/SCID mice. Afterward, metastatic breast cancer in the lung was developed by administering MDA-231 cells into the lateral tail vein of mice of the same strain. hUC-MSC (1 × 10^6^) were administered in the tail vein on days 17 and 24 or 11 and 18 after tumor inoculation to evaluate their selective engraftment. In another experiment, IFN-β-expressing hUC-MSC (0.5 × 10^6^) were administered via tail vein eight days after tumor inoculation twice at 1 week intervals to evaluate their ability to reduce tumor burden. Overall, hUC-MSC do not form tumors, selectively engraft in lung tumors, and reduce the tumor burden in SCID mice following systemic administration [59].

Conversely, some studies have reported that hUC-MSC could present tumorigenic properties in breast cancer models. It was verified that hUC-MSC could lead to tumor progression in a breast cancer model derived from the MCF-7 cell line in BALB/c athymic mice [60]. Also, hUC-MSC do not significantly influence the tumor volume, and weight compared to control but lead to the appearance of sporadic tumor cells in the lungs of some animals. Moreover, some tumor knobs were also observed in the lungs of some animals [60]. In another study, using a CB17 SCID mice model derived from MDA-MB-231 stem cells, these authors demonstrated that injecting two doses of hUC-MSC (1 × 10^6^ or 3 × 10^6^ cells) induced a significant decrease in tumor growth [61]. These two works could indicate that anti- or pro-tumorigenic effects of hUC-MSC are highly dependent on the target cancer cells. Moreover, antitumor effects observed in cancer stem cells derived from MDA-MB-231 triple-negative breast cancer highlight the therapeutic potential of hUC-MSC against a tumor subtype with low successful curative rates.

In another study, hUC-MSC were loaded with NPs carrying doxorubicin as drug vehicles in a BALB/c nude mice model of breast cancer [62]. These cells presented an efficient tumor tropism. Doxorubicin NPs-loaded hUC-MSC induced a significant decrease in tumor volume compared with the control or hUC-MSC without doxorubicin NPs [62].

Another approach has evaluated the effects of IL-6 secreted from senescent hUC-MSC in the proliferation and migration in a model of triple-negative breast cancer in immunodeficient mice. This therapeutic strategy accelerated tumor growth with a significant increase in tumor vascularization [63].

The effects of an IL-6 pre-treatment of hUC-MSC on their phenotype and function and how it could be related to gastric cancer progression have been studied through an animal model of gastric cancer in which animals were treated with hUC-MSC alone or pre-treated hUC-MSC. BALB/c nude mice treated only with hUC-MSC developed significantly greater tumor volumes than the control and the IL-6 pre-treated cells groups, revealing that this pre-treatment abolished the tumor-promoting effects of hUC-MSC in gastric cancer [64].

Wang and collaborators have exploited an old hypothesis that stated that fusion between cancer cells and normal cells could abolish the malignant phenotype. Thus, the authors investigated if fusion between hUC-MSC and esophageal carcinoma cells effectively leads to tumor inhibition. Most animals developed tumors, and the tumors formed in the fusion groups were significantly smaller than in the control groups [65]. This approach was also studied in a gastric cancer model, in which hUC-MSC were fused with human gastric cancer cell lines HGC-27 and SGC-7901. Fused cells lead to increased tumor volumes compared to the control group, which indicates that these hybrid cells could enhance tumor growth due to induction of epithelial–mesenchymal transition (EMT) and stemness of gastric cancer cells [66].

Simultaneous injection of cholangiocarcinoma cells and hUC-MSC in a BALB/c nude mice model revealed a lower tumor incidence compared to the control group. Moreover, injection of the hUC-MSC conditioned media led to a tumor volume decrease compared to the control group. This antitumor effect could be due to the inhibition and downregulation of Akt and Wnt signaling pathways induced by hUC-MSC [67].

The role of Wnt/β-catenin signaling pathways activated by hUC-MSC in the development of cholangiocarcinoma has been exploited [68]. Tumor volume and weight were significantly higher when cholangiocarcinoma cells were injected with hUC-MSC. Thus, hUC-MSC could have a role in cholangiocarcinoma tumorigenesis [68]. Moreover, the authors have also demonstrated a significant increase in the number of metastasis and chemoresistance induced by compound K in the mixed cells group compared to control, which could be due to increased expression of β-catenin and activation of Wnt signaling pathways induced by the hUC-MSC [68].

Also, hUC-MSC can be engineered to overexpress specific proteins that could be interesting antitumor players. The effects of engineered hUC-MSC with sTRAIL gene expression in liver cancer that could secrete cytotoxic ILZ-sTRAIL protein have been investigated to assess its clinical potential combined with 5-FU. Engineered hUC-MSC presented tropism towards hepatocarcinoma cells in an orthotopic mouse model. Tumors treated with engineered hUC-MSC-ILZ-sTRAIL and treated with the combination scheme led to a significant tumor regression compared to the control group [69]. These authors also exploited the potential of this hUC-MSC-ILZ-sTRAIL fused with CD20 to target non-Hodgkin’s lymphoma (NHL) [70]. It was shown that these engineered cells could migrate to the tumor site and specifically release the fusion protein scFvCD20-sTRAIL with decreased tumor growth in the SCID animals treated with this engineered hUC-MSC compared to control group [70].

Also, the effects of transduced hUC-MSC with a vector containing the full-length cDNA sequence of TRAIL under the control of the pIL6 in multiple myeloma were studied in vivo. The animal model was obtained by intratibial injection of 2 × 10^5^ Red-Luc+U-266 cells in the NOD.CB17-Prkdcscid/J mice. Here, hUC-MSC (2.5 × 10^5^) were administered intracardially three days after inoculation of tumor cells. Consequently, tumor burden was significantly reduced by specific induction of apoptosis [71]. A combined approach using hUC-MSC carrying the IL-21 gene and miR-200c was proposed to treat epithelial ovarian cancer in nude mice. IL-21 can induce an antitumor immune response, while the microRNA miR-200c leads to inhibition of EMT. The results showed that tumor growth was significantly reduced after the treatment with hUC-MSC-IL-21 + miR-200c. Moreover, there was no toxicity associated with this treatment approach. It was also proposed that these antitumor effects could be due to the induction of a robust immune response and activation of the Wnt/β-catenin signaling [72]. Furthermore, hUC-MSC were investigated as a vehicle for delivering IL-24 in the treatment of glioma. The BALB/c nude mouse was used to develop the glioma model by subcutaneous administration of 2 × 10^6^ U251 cells into the left flank near the axillary fossa. The hUC-MSC with IL-24 (2 × 10^6^) were administered ten days post tumor cell implantation via tail vein injection every week for three weeks. The hUC-MSC expressing IL-24 induced apoptosis and reduced tumor growth of gliomas in vivo [73].

In a different approach, the efficacy of using genetically modified hUC-MSC, which constitutively secreted Tandab (CD3/CD19), a tetravalent bispecific tandem diabody with two binding sites for CD3 and two for CD19, was explored for the treatment of B cell lymphoma. The BALB/c nude mouse was used to develop a B cell lymphoma model by subcutaneous administration of 2 × 10^7^ Raji cells into the right flank one day after total body irradiation (300 cGy). In the first experiment, hUC-MSC (1 × 10^6^) were injected intravenously, and a selective accumulation was seen at the tumor site 24 h after injection. In a second experiment, the hUC-MSC (1 × 10^6^) were injected intravenously with PBMCs and D-1MT (D-1-methyl- tryptophan) in the drinking water. This combination decreased the tumor weight by 61.2%, demonstrating the potential of hUC-MSC as a cell-based delivery vehicle for treating B cell lymphoma [74].

Intravenous administration of hUC-MSC engineered to express IFN-β decreases tumor formation of human bronchioloalveolar carcinoma cells and induces cell death via both extrinsic and intrinsic apoptotic pathways. An orthotopic animal model of lung cancer was developed by administering H358 cells through the lateral tail vein in CB17/SCID mice. The IFN-β-expressing hUC-MSC (3 × 10^5^) were injected through the tail vein every five days for four times [75]. IFN-β-expressing hUC-MSC significantly reduced tumor burden in vivo, as previously reported [59,75]. Another study also investigated the growth attenuation potential of naïve hUC-MSC in the same metastatic breast cancer in the lung model described above [59]. In this case, hUC-MSC (0.5 × 10^6^) were administered eight days post tumor inoculation for three weeks. The hUC-MSC reached the tumor site and attenuated the tumor growth in the lungs. The authors hypothesize that tumor growth attenuation might be associated with the targeted homing of hUC-MSC to tumor tissue [25]. A different approach using the hUC-MSC engineered to express an endogenous tumor suppressor gene, follistatin, was studied in the same metastatic breast cancer in the lung model used in two other studies described above [25,59]. The follistatin over-expressing hUC-MSC (5 × 10^5^) were intravenously administered 6, 13, and 20 days after the tumor cell’s inoculation. Follistatin over-expressing hUC-MSC attenuated metastatic tumor growth and tumor nodule number in the lung [26].

The use of hUC-MSC as a therapeutic approach for both pulmonary and gastric carcinoma has been exploited. Here, two approaches have been used: injection of hUC-MSC together with tumor cells or 12 days after tumor cells inoculation. Results demonstrated that co-injection led to greater tumor volumes than control (saline administration only) groups and the group of hUC-MSC injected after 12 days. The same results were observed for using human amniotic mesenchymal stem cells [76].

Another potential application of hUC-MSC described is the ability to mitigate the side effects induced by adriamycin while not impacting tumor progression. Two animal models were used, a murine Lewis lung carcinoma model and a human colon carcinoma xenograft model. On the one hand, the C57BL/6 mice were used to develop a murine Lewis lung carcinoma model by subcutaneous administration of 2 × 10^6^ LLC cells in the right flank region, with hUC-MSC (1 × 10^6^) administered intravenously. On the other hand, the BALB/C nude mice were used to develop a human colon carcinoma xenograft model by subcutaneous administration of 5 × 10^6^ Lovo cells into the left flank, with hUC-MSC (0.5–1 × 10^6^) administered intravenously. The hUC-MSC reduced the adriamycin-induced side effects and improved recipients’ general quality of life as an adjuvant therapy during and post-chemotherapy in both animal models [77].

The potential application of hUC-MSC in three other lung cancer models have been explored by applying different approaches. A study has demonstrated that hUC-MSC induced an increase of the tumor growth compared to control in a BALB/c nude mice model derived from the implantation of lung cancer cells H1299 [78]. Moreover, it was also demonstrated in vitro that nicotine can promote hUC-MSC migration, enhance the stem cell properties of hUC-MSC, and induce EMT. These alterations induced by nicotine could trigger the lung cancer progression induced by hUC-MSC in the murine model [79]. The tropism of hUC-MSC-ILZ-sTRAIL towards lung cancer cells was assessed in an orthotopic mouse model. These cells showed ability to migrate to the tumor location only 24 h after administration. Moreover, it was shown that, despite no significant difference in tumor size, the survival rate in the MSC-ILZ-sTRAIL-treated group was higher than in the control group [80].

As initially stated, MSC could have a dual effect in cancer animals’ models by either inhibiting or promoting tumor growth and progression, indicating that the observed effects could be highly dependent on tumor and cell characteristics.

### 7.2. Human Umbilical Cord Wharton’s Jelly Mesenchymal Stem Cells (hUC-WJ-MSC)

Wharton’s jelly has been widely explored in anticancer research, including in animal studies; however, there still are several discrepancies regarding the role of hUC-WJ in carcinogenesis, tumor growth and tropism, and cancer-related inflammation.

The hUC-WJ’s ability to induce tumorigenesis, comparatively to human embryonic stem cells, was addressed by Gauthaman and colleagues [81]. In this study, hUC-WJ harvested from hUC were labeled or not with red fluorescence protein (RFP) and prepared with or without Matrigel for transplantations into 7–8-week-old female SCID mice. Then, each group was injected subcutaneously or intramuscularly, in one to five sites, with 5 × 10^6^ cells of hUC-WJ in saline or Matrigel, a known agent for enhancing in vivo teratoma formation, and animals were maintained for up to 20 weeks. The results showed that transplantation with hUC-WJ is hypoimmunogenic and does not lead to teratomas or tumor development. This demonstrates its safety application for clinical cell-based therapies [81].

The anticancer effects of conditioned medium or human Wharton’s jelly MSC (hUC-WJ-MSC) were studied in vivo in a xenograft immunodeficient mouse model of human mammary carcinomas [82]. The animal model was developed by injecting 1 × 10^6^ MDA-MB-231 cells over the shoulder region in 6–8-week-old SCID mice seeking the development of ectopic breast tumors. Four days (early protocol) or five weeks (late protocol) after mammary carcinoma cell transplantation, 1 × 10^6^ or 5 × 10^6^ of hUC-WJ-MSC, and hUC-WJ-MSC conditioned medium (50%), obtained after 48 h of incubation with cells, were administered intratumorally. Compared to control, a decrease in tumor sizes and weights was observed six weeks after injection of hUC-WJ-MSC and their conditioned medium, with greater tumor attenuation observed in late tumor protocol. This tumor attenuation was associated with lymphocyte infiltrations and vacuolization of tumor cells in the treated groups [82].

Vulcano and colleagues used hUC-WJ-MSC to co-inject with human lung cancer stem cells (LCSC) into female, 6 to 8-week-old NOD/SCID mice. Two types of LCSC were used, one derived from adenocarcinomas (AC) and the other from squamous cell carcinomas (SCC). The co-inoculation of AC-LCSC with hUC-WJ-MSC generated larger tumors than inoculation of AC-LCSC alone. In contrast, the co-inoculation of SCC-LCSC with hUC-WJ-MSC did not affect tumor size. These results suggest that the effects induced by hUC-WJ-MSC depend on the tumors and their subtypes [83].

The possible role of MVs derived from hUC-WJ-MSC in mediating bladder antitumor effects was studied by Wu and colleagues [84]. They developed a xenograft model of bladder tumor in 4-week-old male BALB/c nu/nu mice by subcutaneous injection of 1 × 10^7^ T24 cells or hUC-WJ-MSC alone or simultaneously with 200 µg protein of MVs derived from hUC-WJ-MSC. Results obtained 30 days post tumor inoculation showed that inoculation of tumor cells with MVs derived from hUC-WJ-MSC exerted potent antiproliferative and proapoptotic effects on in vivo bladder tumor in higher magnitude than when inoculated together with hUC-WJ-MSC [84]. Du and colleagues used a similar approach [85] for analyzing the potential of MVs derived from hUC-WJ-MSC in promoting renal cell carcinoma (RCC) growth and invasiveness, with possible application for regenerative medicine. They developed a xenograft model by injecting 1 × 10^7^ cells of a human RCC line (786-0) subcutaneously into BALB/c nu/nu mice, plus MVs released from hUC-WJ-MSC overnight (200 µg/mL), RNase-MVs or control. Results showed that MVs derived from hUC-WJ-MSC can promote RCC growth and invasiveness by activating AKT and ERK1/2 signaling. The effects induced by MVs were nullified when pretreatment with RNase was performed, indicating the role of RNA information present in MVs in mediating this process. To elucidate if antitumor effects can depend on the MSC sources from which EVs are derived and on the cancer type, Mirabdollahi et al. evaluated whether EVs derived from hUC-WJ-MSC could stimulate or inhibit the in vivo growth of breast cancer [86]. The hUC-WJ-MSC, isolated from the human healthy pregnancies’ umbilical cords, were used to isolate the secretome produced during cell culture in a serum-free medium. In vivo breast cancer model was developed in 4–6 weeks old female BALB/C mice. Then, the animals were split into three groups, three for each approach. In the first approach, mice were treated with three intravenous injections of hUC-WJ-MSC-derived secretome, Cisplatin, and PBS, on days 5, 10, and 15, and then, on day 30, the mice were inoculated with 3.5 × 10^6^ of 4T1 cells. In the second approach, mice were first subcutaneously injected with 3.5 × 10^6^ 4T1 cells. When the tumor appeared, they received three injections of hUC-WJ-MSC-derived secretome, Cisplatin, and PBS at five-day intervals. Administration of hUC-WJ-MSC-derived secretome before cancer induction (preventive model) showed significant anticancer activity against breast cancer. The tumor-bearing mice treated with hUC-WJ-MSC-derived secretome induced slower tumor progression, smaller tumor size and weight, more extended latency period, and prolonged survival rate [86].

The use of hUC-WJ-MSC and biological products could constitute a candidate for cancer therapy, considering the non-tumorigenic and antitumorigenic properties demonstrated [83,84,85,86]. More recently, the potential of hUC-WJ-MSC for a theragnostic purpose has also been sought. The labeling of hUC-WJ-MSC with Mn^2+^ and Gd^3+^ co-doped CuInS2-ZnS (CIS-ZMGS) NCs has been experimented with by Chetty and colleagues [87] for two hours for posteriorly multi-modality imaging. For this in vivo imaging experiment, adult C57BL/6 mice were inoculated with 105 B16F10 melanoma cells on the shoulder and left hind limb towards skeletal muscle to develop tumors. Once the tumor reached the desired growth, 16F10 tumor-bearing C57BL/6 mice models were injected into the tail vein with CIS-ZMGS NCs labeled hUC-WJ-MSC (106) and imaged for six hours. Labeling these cells did not affect their differentiation, immune phenotypes, proteins, or gene expression. Moreover, there was a positive tumor tropism through this modality, showing the potential of using imaging techniques to diagnose early melanoma [87].

### 7.3. Human Umbilical Cord Perivascular Cells (hUC-PVC)

The perivascular region comprises almost 45% of the cells in Wharton’s jelly. These hUC-PVC are positive for mesenchymal stromal cell markers [27]. Therefore, hUC-PVC conditioned media effects on tumor growth and angiogenesis were studied. The Chick Chorioallantoic Membrane assay was used to develop an in vivo 3D model of glioblastoma by administering 2 × 10^6^ U251 or SNB-19 cells on a window made into the eggshell after puncturing the air chamber. These tumor cells were previously exposed to hUC-PVC conditioned media for four days. The results revealed that previous exposure to hUC-PVC conditioned media resulted in significantly larger tumors and increased vessel densities in both U251 and SNB-19 cells. Overall, hUC-PVC secret molecules that contribute to higher glioblastoma tumor growth in vivo [88].

### 7.4. Human Umbilical Cord Mesenchymal Stem Cells-Derived Extracellular Vesicles (hUC-MSC-EV)

Extracellular vesicles (EVs) are released in high quantity by MSC and serve as paracrine mediators. EVs are small membranous vesicles whose diameter varies between a few micrometers to 50–100 nanometers and can be classified as exosomes, microvesicles (MVs), ectosomes, oncosomes, and apoptotic bodies, mediating cell-to-cell communication and transfer of biological material to adjacent or distant targets [78,89]. EVs could actively deliver microRNAs (miR), either constitutive or pre/loaded molecules [90]. The study of miRs in oncology has gained momentum, as miRs can serve as tumor-suppressing or tumor-promoting mediators [90]. Considering the tumor-suppressing properties offered by some miRs, a recent study assessed the potential of miR-320a-containing hUC-MSC-derived exosomes in a BALB/c nude xenograft model inoculated with H1299 lung cancer cells. The authors measured a significant decrease in tumor growth and a reduced expression of SOX4, Wnt1, and β-catenin in tumor tissues [91].

Using hUC-MSC-derived exosomes transfected with miR-375 led to decreased tumor growth in a murine model of esophageal squamous cell carcinoma [92]. Moreover, there was elevated expression of miR-375, BAX, and E-cadherin. There was a decrease in the expression of ENAH, Bcl-2, Bcl-x, N-cadherin, and Snail in tumor tissues of the group treated with hUC-MSC-EV transfected with miR-375 compared to control animals [92]. Similarly, Jia et al. studied the transfer of miR-139-5p from hUC-MSC-EV to bladder cancer in vivo and their role in tumorigenesis, showing that the volume and weight of tumors treated with hUC-MSC-derived exosomes were reduced compared to control animals [93].

In a breast cancer mouse model, miR-148b-3p from exosomes derived from hUC-MSC led to a remarkable decrease in tumor volume and weight compared to the control group, due to TRIM59 inhibition by miR-148b-3p. Moreover, tumors from animals treated with miR-148b-3p of hUC-MSC-EV showed a decreased expression of Ki-67, N-cadherin, and Vimentin [94].

The effects of miR-30c-5p-carrying-hUC-MSC-EV in a mouse model of papillary thyroid carcinoma were studied. It was demonstrated that tumor volume and weight were significantly decreased after administration of miR-30c-5p-carrying-hUC-MSC-EV compared to the control group, which could be due to the inhibition of E3 ubiquitin ligase Pellino-1 (PELI1) by miR-30c-5p. Moreover, it was also demonstrated that naïve hUC-MSC-EV inhibited papillary thyroid carcinoma in vivo [95].

In addition, it was demonstrated that exosomes derived from hUC-MSC accumulate in the tumor site for 24–48 h after intravenous injection in an in vivo mouse model of osteosarcoma. An immunodeficient nu/nu mouse was used to develop an ectopic osteosarcoma model by subcutaneous injection of 1 × 10^6^ K7M2 cells in the lower flank of the mouse. The exosomes labeled with gadolinium were injected ten days after implantation of tumor cells through the lateral tail vein, and the biodistribution was evaluated 24 h post-injection. In the tumor, the accumulation of the exosomes reached 18%, higher than the commercially available control used, Magnevist^®^, an approved standard MRI contrast agent used to facilitate the visualization of the lesion and abnormal vascularity in the body [96].

In another study, the hUC-MSC-derived exosomes were used to deliver, in vivo, a tumor suppressor that is frequently downregulated in pancreatic ductal adenocarcinoma, the miR-145-5p. The BALB/c nude mouse was used to develop an ectopic pancreatic ductal adenocarcinoma model by subcutaneously injecting 1 × 10^6^ PANC-1 cells on both flanks of the mice and the exosomes were administered intratumorally. A decrease in tumor growth and downregulation of Smad3, N-cadherin, and Bax expression and significant upregulation of E-cadherin and Bcl-2 expression were observed after administering the exosomes [97].

On the other hand, it was demonstrated that isolated exosomes derived from the hUC-MSC increased tumor growth [78]. Further investigation concluded that this effect was due to the transfer of miR-410 from hUC-MSC exosomes to lung cancer cells [78].

The delivery of miR-224-5p by exosomes derived from hUC-MSC led to higher tumor growth than in the control group. In addition, the delivery of an inhibitor of miR-224-5p significantly decreased the tumor volume and weight compared to control in a nude mice model of breast cancer, which could be due to HOXA5 downregulation by miR-224-5p and consequently modulating autophagy [98].

The injection of exosomes derived from hUC-MSC promoted the tumor growth of pancreatic tumor cells PANC-1 in an animal model compared to control, which could be due to the transferring of miR-100-5p from hUC-MSC to pancreatic cancer cells [99].

The effects of hUC-MSC-derived exosomal miR-181a in a murine animal model of nasopharyngeal carcinoma have been assessed. miR-181a was downregulated in nasopharyngeal carcinoma cells; thus, hUC-MSC could deliver this miR to tumor cells. In vivo studies revealed that injection of exosomes derived from hUC-MSC led to decreased tumor volume and weight compared to control. Moreover, when injecting exosomes from miR-181a inhibitor-transfected hUC-MSC, an increased tumor proliferation was observed [100].

## 8. Discussion

The evidence summarized and described in this narrative review support possible anticancer effects offered by human PnD cell or tissue products. However, critical results support risky tumor-promoting effects emanating from similar PnD products. In vitro studies reporting this dichotomous role in perinatal MSC have been identified and described [19]. These opposing effects were also reported in the animal studies revised here and may be attributable to different factors: (1) variability of the PnD used; (2) different PnD isolation methods; (3) huge variability of experimental designs, particularly concerning dosage, route, and time of administration, and time of evaluation; (4) the animal models; and also (5) tumor types and characteristics.

Figure 1 summarizes all the different PnD studied for their anticancer effect. Experimental details on the methodology of tissue collection and PnD isolation and preservation are often sparse or even absent. Unfortunately, many reports provided scarce information or details about the donor (e.g., ethnicity, age, gestational age, infections, significant lifestyle factors, relevant pre-existing medical conditions), and the proper characterization of the PnD product tested (e.g., phenotypical, genetic, functional). Several preclinical studies here analyzed include different PnD products, such as tissue extracts, minimally manipulated or engineered cells, conditioned media, and/or purified EVs. The high variability in PnD approaches and the scarce information hamper data comparability and reproducibility. A previous systematic review published within the COST SPRINT Action (CA17116) proposed a list of guidelines for an adequate characterization of PnD before their use in preclinical models, which can be almost entirely transposed to the oncology field [7].

The discrepancy within study designs represents another limitation to PnD validation studies for oncological treatments, both in terms of application parameters (time points for administration, effect evaluation, repeated or single administration events, cell dose, route of administration), as well as in clear, structured outcome evaluation. The use of several approaches for each of these parameters creates high heterogeneity between studies and hampers the comparability of the results. Besides, an inaccurate description of the animal models and experimental protocols also hinders the studies’ reproducibility and the opportunity to conclude the anticancer potential of different PnD strategies. A standardized, human-relevant animal model and accurate description are imperative.

Animal models are a valuable (currently) irreplaceable model for studying pathophysiology in cancer research, critical for the discovery and preclinical validation of new oncological therapeutic approaches [101]. Preclinical models’ main advantages rely on the recapitulation of tumor initiation and progression in a more pathophysiologic environment [102,103]. In general, rodent cancer models are the most frequently used and include xenografts and chemically or genetically induced cancers. All the studies reviewed here except one performed xenotransplantation, where human PnD products were implanted or infused in animals. Although heterotopic models are inexpensive, are easy to obtain, and allow the follow-up of tumor development, the correlation to clinical response is limited. This disadvantage is surpassed in orthotopic models since cells are implanted in the tumor microenvironment [101]. Only one study reported an orthotopic model within the revised study cohort. Subcutaneous heterotopic models are an excellent tool for the first screening of antitumor activity of PnD in vivo. However, it is essential to use models able to mimic the stroma–tumor–PnD interactions and prove PnD effectiveness in the proper tumor microenvironment and with adequate vascularization. Further, mice or rats described are immunocompromised not to reject human PnD products, such as athymic nude mice or severely compromised immunodeficient (SCID) mice. This fact constitutes an additional limitation for evaluating the anticancer effects of PnD-based cell therapy, knowing in advance that perinatal cells have a strong immunomodulatory potential [12], which cannot be assessed in these models. One study reported using a non-rodent animal model, the chicken chorioallantoic membrane assay. This model presents several advantages: it is rapid and cost-effective, it recapitulates many tumors’ characteristics, allows study of angiogenesis, migration, and invasion, and assesses the efficacy of anticancer drugs [104].

**Figure 2:**
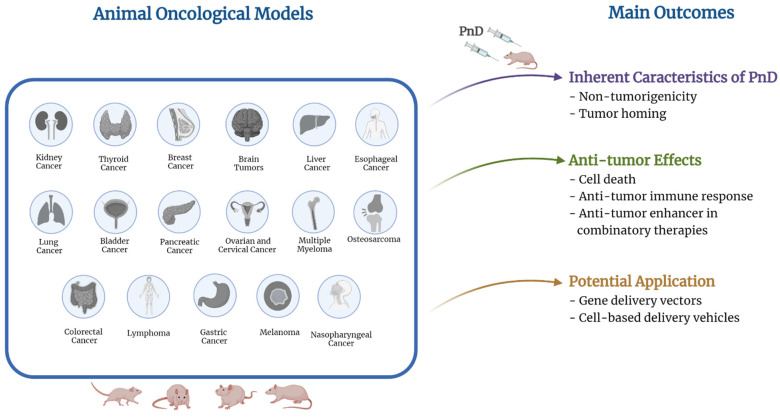
Summary of the animal oncological models for which the anticancer effects of PnD have been assessed and the main outcomes observed.

The herein described animal studies evaluated the efficacy of different PnD towards different types of cancer (Table 1, Figure 2), namely kidney cancer [85], pancreatic cancer [97,99], ovarian cancer [36,46,47,49,72], breast cancer [25,26,28,29,40,41,43,51,52,58,60,61,62,63,64,83,87,95,99], osteosarcoma [96], multiple myeloma [58,71], lung cancer [41,44,75,77,78,79,80,83,91], liver cancer [34,38,54,55,67,68,69], brain tumors [35,43,53,56,73,88], colorectal cancer [24,33,37,50,77], bladder cancer [45,84,93], melanoma [23,50,52,87], cervical cancer [48], lymphoma [70,74], gastric cancer [32,64,66], nasopharyngeal cancer [100], esophageal cancer [65,92], and thyroid cancer [95].

Despite the limited number of studies published so far, unable to draw solid conclusions, it is important to account that some authors reported undesired tumor-promoting effects as a result of PnD implantation (Table 1, Figure 2), namely tumor development through activation of signaling pathways involved in tumor progression (e.g., AKT, ERKT1/2). Furthermore, additional effects have been reported favoring CSC phenotype and chemoresistance, or negative outcome has been associated with the transfer of miRs from PnD-derived exosomes to cancer cells. The majority of the studies reported several PnD anticancer benefits (Table 1, Figure 2): (1) tumor homing ability; (2) non-tumorigenicity; (3) tumorigenesis prevention; (4) inhibition of tumor growth or regrowth through suppression of angiogenesis, induction of cell death, an increase of pro-apoptotic factors, decreased expression of anti-apoptotic factors, downregulation of signaling pathways involved in tumor progression, inhibition of EMT, elevated expression of tumor-suppressing miRs, and lymphocytic infiltrations; (5) antitumor immune response; (6) effective gene delivery vectors; (7) effective cell-based delivery vehicles of NPs or antitumor factors, and (8) antitumor response enhancers in combinatory therapeutic approaches.

In summary, applying PnD in oncology is far from being included in clinical practice. Although scientific evidence indicates that PnD isolated from the placenta, amniotic membrane, amniotic fluid, or umbilical cord may have anticancer potential, the paradoxical antitumor and pro-tumor effects exist and must be clarified. The safe use of PnD against cancer depends on the joint efforts of all researchers in following proper guidelines and accurately describing the methodology of tissue collection, PnD isolation, preservation, and experimental design.

## Figures and Tables

**Table 1:** In vivo studies of PnD application on oncological animal models.

Reference	Disease Target	Animal Model	PnD	PnD Subtype	Experimental Design	Outcome
Park, J. M. et al., 2021	Gastric Cancer	C57BL/6 mice; intragastric inoculation of Helicobacter pylori	hPC	hPMSC	22 weeks after Helicobacter pylori infection, oral administration (about ten times) of 1 × 10^7^ hPMSC or 100 μL of concentrated conditioned medium from hPMSC	Significant reduction of inflammation and gastric atrophy, which can contribute to the prevention of the evolution of Helicobacter pylori-associated gastric precancerous lesions to gastric cancer.
Ma, X. et al., 2020	Colon cancer	BALB/c nude mice; SC injection of HCT116-GFP cells	hPC	hPMSC	When tumors reached 50 mm^3^, administration in the tail vein of 1 × 10^6^ hPMSC, every 4 days, four administrations	Tumor development induction CSC phenotype promotion Increase in the CD133 expression
Hajighasemlou, S. et al., 2018	Hepatocellular carcinoma	C57BL/6 nude mice; SC injection of HepG2 cells	hPC	hPMSC	15 days after tumor inoculation, IV injection in tail vein and tumor margins of 5 × 10^5^ hPMSC	Higher ability for tumor tropism
Hsu, F. T. et al., 2018	Glioblastoma	BALB/c nude mice; U87 GSCs injection 2 mm below the brain surface	hPC	hPMSC	Two weeks after tumor inoculation, IV injection into tumor-bearing mice and IV and IP injection into normal mice of hPMSC	Rapid clearance of hPMSC hPMSC tropism to glioblastoma
Chen, Q. et al., 2012	Melanoma	C57BL/6 mice; SC injection of B16-F10 melanoma cells	hPC	hPMSC	When the tumor reached 3 mm diameter, two IT injections of 5 × 10^5^ hPMSC, 4 days apart	Significant reduction of tumor volume and cell apoptosis by hPMSC expressing PEDF
Zheng, L. et al., 2012	Ovarian cancer	BALB/c nude mice; IP injection of A2780 cells	hPC	hPMSC	Homing study: 16 days after tumor inoculation, IP injection of 2 × 10^5^ hPMSC Treatment study: 5 days after tumor inoculation, IP injection of 2 × 10^5^ hPMSC, every 3 days, six times	Inhibition of tumor development, angiogenesis Promotion of tissues apoptosis Tumor homing capacity
Zhang, D. et al., 2014	Colorectal cancer	BALB/c mice; CT26 cells injection into the abdomen cavity	hPC	hPMSC	Four days after tumor implantation, IP injection of 1 × 10^5^ of hPMSC, every 4 days, four administrations	Inhibition of tumor development and angiogenesis Induction of tumor apoptosis
Yang, J. et al., 2019	Colon cancer	Nude mice; SC injection of HT29 cells (HT29-DF) transfected with DF gene (Flu-eGFP) in the right axilla	hPC	hPMSC	Ten days post tumor cells implantation, IV injection into the tail vein of 200 µL of transduced hPMSC	Engineered hPMSC-DF: tumor growth inhibition; tumor tropism capacity hPMSC-DF + GCV: tumor growth inhibition; induction of apoptosis of tumors
Seyhoun, I. et al., 2019	Hepatocellular carcinoma	BALB/c nude mice; SC injection of HepG2 cells into both flanks	hPC	hPMSC	When tumors reached a volume superior to 100 mm^3^, IT injection of 5 × 10^5^ hPMSC 4 times, 3 days apart	Inhibition of tumor spreading and apoptosis by hPMSC + sorafenib No effect induced by hPMSC alone
Kamalabadi-Farahani, M. et al., 2018	Triple-negative breast cancer	BALB/c nude mice, syngeneic animal model; SC injection of 4T1 cells into the flank	hPC	hPMSC	Seven days after tumor implantation, injection in the tumor area of 1 × 10^6^ of each type of hPMSC	Inhibition of tumor proliferation and apoptosis by combination therapy No effects with hPMSC alone
Allen, H. et al., 2018	Triple-negative breast cancer	Foxn1^nu^ nude mice; SC injection of MDA-MB-231 cells into the right flank and injection into the left inguinal mammary fat pad	hPC	hP-ASC	Heterotopic model: on days 9 and 28 after tumor cells implantation, IM injection of 1 × 10^6^ and 5 × 10^6^ induced hP-ASC; on day 28, IM injection of 5 × 10^6^ non-induced hP-ASC. The control group was untreated mice. Orthotopic model: from day 48 to 83, weekly injection of 1 × 10^6^ induced hP-ASC. The induced hP-ASC group from day 6 to 41 received PlasmaLyte weekly prior to administration of hP-ASC. The control group received PlasmaLyte weekly from day 6 to 83	Slower tumor progression in orthotopic animals after treatment with induced hP-ASC Complete response in 30% of treated animals
Li, L. et al., 2015	Lung cancer	BALB/c nude mice; SC injection of A549 cells	hAF	hAFSC	After tumors reach a volume of 150 mm^3^, IV injection of 5 × 10^6^ cells per day for two consecutive days	Tumor homing capacity Impaired tumor growth with increased necrosis by hAFSC overexpressing DAL-1 No hAFSC mRNA in liver, lung, spleen, heart, kidney, small intestine, and testis
Kang, N. H. et al., 2012	Breast cancer	BALB/c nude mice; SC injection of MDA-MB-231 cells on mammary fat pads	hAF	hAFSC	Seven weeks after tumor inoculation, circumtumoral injection of 4 × 10^6^ cells two weeks apart	Migration of hAFSC to IT region Inhibition of tumor growth after treatment with hAFSC expressing suicide genes + 5-FC + GCV No toxicity in breast tissues induced by hAFSC High toxicity in breast tissues induced by 5-FU
Li, L. et al., 2015	Ovarian cancer	Nude mice; (1) SC injection of SKOV3 cells or hAFMSC, (2) SC injection of SKOV3 cells	hAF	hAF-MSC	(1) At the beginning of the study, SC injection of 6 × 10^6^ cells (2) A week after ovarian cancer cell inoculation, IV injection of 6 × 10^6^ cells, two weekly injections	(1) No tumor formation (2) Tumor tropism capacity, with cells detected in the liver and spleen
You, Q. et al., 2015	Ovarian cancer	(1) SCID mice; IM injection of hAF-MSC (2) BALB/c nude mice SC injection of SKOV3 cells	hAF	hAF-MSC	(1) At the beginning of the study, IM injection of 3 × 10^6^ cells (2) When tumors reached 1 cm in diameter, IV injection of 4 × 10^6^ cells	(1) No tumor formation (2) Tumor tropism capacity
Bitsika, V. et al., 2012	Bladder cancer	NOD-SCID mice; SC injection of T24M cells near the tail base	hAF	hAF-MSC	Ten days after tumor inoculation, an IV injection of 1 × 10^6^ cells was administered in three weekly doses	hAF-MSC migration to tumors No changes in tumor growth by hAF.MSC Inhibition of tumor growth by hAF-MSC carriers of IFNβ Small number of hAF-MSC was found in the lungs
Zhou, J. et al., 2018	Cervical cancer	BALB/c nude mice; SC injection of eGFP-HeLa cells	hAF	hAF-MSC	Ten days after tumor inoculation, IV injection of 5 × 10^6^ hAF-MSC cells. Three doses of hAF-MSC (naif vs. overexpressing IFNα) doses every five days or a single administration (biodistribution).	Tumor homing capacity of hAF-MSC Few cells were found in the liver and spleen Inhibition of tumor growth after three administrations of IFNα overexpressing hAF-MSC Increased tumor growth induced by naive hAF-MSC
Yin, J. et al., 2011	Glioma	BALB/c nude mice; (1) Stereotactic injection into the brain, (2) SC injection of U87MG-EGFRvIII cells	hAF	hAF-MSC	(1) Stereotactic injection of 5 × 10^4^ cells, simultaneously with glioma cells. (2) Injection in the resection cavity of the tumor of 2.5 × 10^5^, when tumors reached 2 cm^3^, after removal of 90% of the tumor mass	No tumor formation after hAF-MSC (alone) orthotopic injection Reduction of tumor burden after treatment with hAF-MSC carrying endostatin and carboxylesterases Inhibition of tumor regrowth after treatment with hAF-MSC expressing endostatin and carboxylesterase
Du, J. et al., 2019	Lung cancer	BALB/c and NOD-SCID mice; SC injection of H460 cells	hAF	hAF-MSC	SC injection of 2 × 10^6^ cells for BALB/c animals and 1 × 10^6^ or 2 × 10^6^ for NOD-SCID mice, simultaneously with cancer cells	Promotion of the earlier onset and delayed the disappearance of tumor mass by hAF-MSC or IFN-γ-primed hAF-MSC Delayed onset and promotion of the disappearance of tumor mass by IFN-β- or IFN-β + IFN-γ-primed hAF-MSC
Tabatabaei, M. et al., 2018	Colorectal, melanoma, and breast cancer	BALB/c and C57BL/6 mice; SC injection of CT26 (colon), Renca (kidney), 4T1 (breast) or B16F10 (melanoma) cells	hAM	hAEC	At the beginning of the study, SC injection of 1 × 10^6^ hAEC, three weakly administrations	Complete inhibition of colorectal tumor development No alteration induced in breast cancer Delayed melanoma tumor development and reduced tumor weight Increased percentage of peripheral blood and splenic T cells (CD3+) in hAEC and CT26 vaccine groups Increased frequency of peripheral and splenic CD4+ T and CD8+ cells Increased activity of splenocytes activity against CT26 cells from hAECs-vaccinated mice Increased level of IFN-γ after stimulation with hAECs or CT26.
Bu, S. et al., 2017	Ovarian cancer	BALB/c nude mice; SC injection of SK-OV-3 cells or SK-OV-3/hAECs	hAM	hAEC	SC injection of 1 × 10^6^, simultaneous with cancer cells	Inhibition of tumor growth, with small tumor size, weight Decreased expression of PCNA and Ki-67
Kang, N. H.et al., 2012	Breast cancer	BALB/c nude mice; SC injection of MDA-MB-231 cells	hAM	hAEC	Seven weeks after tumor implantation or when tumor volumes reached 250–300 cm^3^, circumtumoral injection of 4 × 10^6^ or 8 × 10^6^ cells	Reduced tumor volumes Tumor homing capacity Protection of breast tissues in hAEC group Destruction of breast tissues in 5-FU group Increased survival after hAEC treatment
DiGermano, C. et al., 2016	Melanoma	C57BL/6J mice; SC injection of B16F10 cells	hAM	hAEC	SC injection of B16F10 melanoma cells or hAEC alone or a mix of both cells with increasing amounts of hAECs (0.25–1 × 10^6^)	Delayed tumor growth with decreased tumor size, but not with tumor weight.
Jiao, H. et al., 2012	Glioma	BALB/c nude mice; SC injection of C6 cells	hAM	hAMSC	Six and twelve days after tumor inoculation, IT injection of 2 × 10^6^ cells in a single dose or three doses, three days	Reduced tumor size after a single administration Increased reduction of tumor volume with multiple doses Increased apoptosis and expression of caspase 3, caspase 8, and BAX/BCL-2 ratio
Mamede, A.C. et al., 2015	Hepatocellular carcinoma	BALB/c nude mice; SC injection of HuH7 or HepG2 cells	hAM	hAMTE	When the tumors reached 300 mm^3^, an IP injection of 60 mg/kg of hAMTE was administered every two days for 12 days	Decreased tumor volume of HepG2 hepatocellular tumors No alterations in HUH7 hepatocellular tumors
Vegh et al., 2012	Breast cancer	Sprague–Dawley female rats; Induction of mammary carcinomas by IP inoculation of NMU (5 mg per 100g body)	hD	hDMSC	When breast tumors were palpable, IV injection of 1.5 × 10^6^ fluorescence-labeled cells	Specific tropism and homing to mammary tumors
Paris et al., 2016	Breast cancer	Sprague–Dawley female rats; Induction of mammary carcinomas by IP inoculation of NMU (5 mg per 100g body)	hD	hDMSC	After tumor development, IV injection of 10^6^ of hDMSC labeled with green fluorescent mesoporus silica NPs	No alterations in tumor homing capacity after NP loading
Yun, J. W. et al., 2016	Hepatocellular carcinoma	A 26-week tumorigenicity study using BALB/c nude mice treated with hUC-MSC	hUC	hUC-MSC	At the beginning of the study, IV injection of 1 × 10^8^ cells/kg, 2 × 10^7^ cells/kg, or 4 × 10^6^ cells/kg of body weight	No tumor formation due to injection of hUC-MSC
Fan, C. et al., 2013	Glioma	Sprague–Dawley mice; Stereotactic injection of C6 cells	hUC	hUC-MSC	One week after tumor cell implantation, contralateral ventricular and IT injection of 5 × 10^5^ cells at 1.3 mm posterior to bregma, 3 mm left to the midline, and 3.5 mm beneath the dura	Migration to glioma site through corpus callosum after contralateral ventricle injection, located at the tumor-normal brain parenchyma interface Distribution at the border zone between tumor and tumor bed after intratumor injection and migration to outgrowing glioma satellites
Ciavarella et al., 2015	Multiple myeloma	NOD.CB17-Prkdcscid/J mice; SC injection of RPMI-8226 cells	hUC	hUC-MSC	2 × 10^6^ cells SC injected simultaneously with the tumor cells or PT injection after 7 days	Tumor inhibition by 50% after simultaneous injection of hUC-MSC Tumor size decreased by 20 times after PT inoculations, 30 days post-implantation of tumor cells
Rachakatla et al., 2007	Metastatic breast cancer in the lung	CB17/SCID mice; IV injection of MDA 231 cells	hUC	hUC-MSC	SC: 2 × 10^6^ or 1 × 10^7^ cells, IV: 2 × 10^6^ or 3 × 10^6^ or 6.5 × 10^6^ cells to evaluate the tumor formation; IV: 1 × 10^6^ cells on days 17 and 24 or 11 and 18 after tumor inoculation to evaluate selective engraftment; IV: 0.5 × 10^6^ cells eight days after tumor inoculation twice at 1-week intervals to evaluate the ability to reduce tumor burden	No tumor induction by hUC-MSC Tumor homing capacity Reduced tumor burden in SCID mice following systemic administration
Ma, F. et al., 2015	Breast cancer	BALB/c nude mice; SC injection of MCF-7 cells	hUC	hUC-MSC	When the tumor reaches 50 mm^3^, IV injection of cells 4 × 10^4^, 2 × 10^5^, 1 × 10^6^ of hUC-MSC	Similar tumor growth with or without hUC-MSC Induction of tumor in the lungs
Ma, Y. et al., 2012	Breast cancer	Nude mice and CB17 SCID mice; Injection of MDA-MB-231 breast CSC into the mammary pad on the right side of the chest wall	hUC	hUC-MSC	When the tumor reaches 0.5 cm, SC injection near the tumor site with 0.5 × 10^6^, 1 × 10^6^, and 3 × 10^6^ cells hUC-MSC, once a week for three consecutive weeks.	Decreased tumor weight dependent on the number of hUC-MSC Decreased expression of PI3K and Akt with the increasing number of hUC-MSC injected
Cao, S. et al., 2018	Breast cancer	BALB/c nude mice; IM injection of 1.5 mg/kg estradiol followed by SC injection of MCF-7 cells into the mice’s armpit	hUC	hUC-MSC	When tumors reached 300 mm^3^, IV injection of 2.5 mg/kg and then every four days	Efficient tumor targeting. Decreased tumor growth induced by doxorubicin nanoparticles-loaded hUC-MSC compared to control and hUC-MSC alone
Di, G. H. et al., 2014	Breast cancer	Female immunodeficient mice; SC injection of MDA-MB-231 alone or mixed with an equal number of hUC-MSC	hUC	hUC-MSC	SC injection of 2 × 10^6^ of hUC-MSC or H_2_O_2_-induced hUC-MSC together with tumor cells, in the right flank region	Increased tumor formation and tumor growth after injection of senescent hUC-MSC Increased vascularization
Wang, M. et al., 2014	Gastric cancer	BALB/c nude mice; SC injection with untreated SGC-7901 cells alone, SGC-7901 cells together with hUC-MSCs or IL-6-pre-treated hUC-MSCs into the backside of mice	hUC	hUC-MSC	At the beginning of the study, SC injection of hUC-MSC	Tumor growth increased in the co-injection group Decreased tumor cell apoptosis in hUC-MSC group. Increased tumor cell apoptosis in IL-6-hUC-MSC group.
Wang, Y. et al., 2011	Esophageal cancer	SCID mice and BALB/c nude mice	hUC	hUC-MSC	At the beginning of the study, injection of 1 × 10^6^ fusion cells for BALB/c nude mice and 1 × 10^5^ for SCID animals	Decreased tumor growth in fusion groups in SCID model; No tumor formation with hUC-MSC alone or self-fused; Increased tumor latency time and decreased tumor weight and volume in BALB/c models with the injection of fused-cells
Xue, J. et al., 2015	Gastric cancer	BALB/C nude mice; SC injection of HGC-27 alone or HGC-27- hUC-MSC fusion cells	hUC	hUC-MSC	At the beginning of the study, SC injection of 2 × 10^6^ hUC-MSC	Increased tumor growth in the fusion-cell group Increased heterogeneity, abnormal nuclear/cytoplasmatic ratio, and derangement distribution in tumor regions
Liu, J. et al., 2013	Cholangiocarcinoma	BALB/c nude mice; SC injection of HCCC-9810 cells or hUC-MSC, or a mixture of cancer cells with hUC-MSC or HUVEC	hUC	hUC-MSC	At the beginning of the study, SC injection of 1 × 10^6^ hUC-MSC, alone or with tumor cells	Decreased tumor incidence with hUC-MSC Decreased tumor volume with hUC-MSC and with hUC-MSC conditioned medium
Wang, W. et al., 2015	Cholangiocarcinoma	BALB/c nude mice; SC injection of QBC939 cells	hUC	hUC-MSC	At the beginning of the study, SC injection of 0.5 × 10^6^ hUC-MSC	Increased tumor volume and weight in the mixed-cell group and hUC-MSC treated group
Yan, C. et al., 2014	Hepatocellular carcinoma	BALB/c nude mice; Orthotopic injection of HepG2 cells	hUC	hUC-MSC	Seven days after tumor implantation, IV injection of 3 × 10^5^ hUC-MSC, 5-FU injected for successive 5 days (10 mg/kg) from the next day of hUC-MSC injection	Tumor tropism capacity Decreased tumor growth by engineered hUC-MSC Synergistic antitumor effects with combination treatment 5FU + engineered hUC-MSC.
Yan, C. et al., 2013	Non-Hodgkin B-cell lymphoma	NOD/SCID mice; SC injection of BJAB cells	hUC	hUC-MSC	One week after tumor cells implantation, an IV injection of 5 × 10^5^ hUC-MSC	Efficient accumulation of fusion protein scFvCD20-sTRAIL secreted by hUC-MSC Tumor growth inhibition by fusion proteins MSC.scFvCD20-sTRAIL and MSC.ISZ-sTRAIL Increased apoptosis in tumor cells, with MSC.scFvCD20-sTRAIL exhibiting greater antitumor potential than MSC.ISZ-sTRAIL.
Cafforio et al., 2017	Multiple myeloma	NOD.CB17-Prkdcscid/J; Intratibial injection of Red-Luc + U-266 cells	hUC	hUC-MSC	Three days after tumor inoculation, 2.5 × 10^5^ cells were injected intracardially	Tumor tropism to multiple myeloma tibia lesions Reduced tumor burden by induction of apoptosis
Zhang, Y. et al., 2018	Ovarian cancer	BALB/c nude mice; SC injection of SKOV3 cells at the mouse’s right flank.	hUC	hUC-MSC	8–9 days after tumor cell implantation, IT injection of 1 × 10^6^ hUC-MSC	Decreased tumor volumes with hUC-MSC-LV-IL-21 combined with miR-200c agomir No evidence of cancer metastasis in the lung, liver, spleen, and stomach Decreased expression of β-catenin, cyclin-D1, Gli1, Gli2, and ZEB1 in the combination group
Fan et al., 2020	Glioma	BALB/c nude mice; SC injection of U251 cells into the left flank near the axillary fossa	hUC	hUC-MSC	Ten days post tumor cells inoculation, IV injection of 2 × 10^6^ cells, every week for 3 weeks	Induction of apoptosis by hUC-MSC expressing IL-24 Reduced tumor growth
Zhang et al., 2017	B cell lymphoma	BALB/c nude mice; SC injection of Raji cells into the right flank	hUC	hUC-MSC	1 × 10^6^ cells IV at day 0; 1 × 10^6^ cells IV at day 0 with PBMCs; IV at day 2 every 7 days for 2 weeks; D-1MT in the drinking water for 21 days	Tumor homing capacity Decreased tumor weight by 61.2%
Matsuzuka et al., 2010	Lung cancer	CB17/SCID mice; IV injection of H358 cells	hUC	hUC-MSC	One week after the second injection of tumor cells, IV injection of 3 × 10^5^ cells, every 5 days, for 4 times	Reduction of tumor burden by IFN-β-expressing hUC-MSC
Ayuzawa et al., 2009	Metastatic breast cancer in the lung	CB17/SCID mice; IV injection of MDA 231 cells	hUC	hUC-MSC	Eight days after tumor implantation, IV injection of 0.5 × 10^6^ cells for 3 weeks	Tumor homing capacity Decreased tumor growth in the lungs
Ohta et al., 2015	Metastatic breast cancer in the lung	CB17/SCID mice; IV injection of MDA 231 cells	hUC	hUC-MSC	On days 6, 13, and 20 after cancer cell inoculation, IV injection of 5 × 10^5^ cells IV, for 4 weeks	Decreased metastatic tumor growth by FST over-expressing cells Decreased number of tumor nodules in the lung
Meng, M. Y. et al., 2019	Lung and Gastric cancer	BALB/c nude mice; IV injection of cells	hUC	hUC-MSC	At the beginning of the study, SC injection of 1 × 10^6^ hAF-MSC and hUC-MSC, mixed with tumor cells	Induction of increased tumor size by hAF-MSC No differences induced by hUC-MSC
Di et al., 2012	Murine Lewis lung carcinoma and human colon carcinoma	C57BL/6 mice; SC injection of cells in the right flank region; BALB/C nude mice; SC injection of Lovo cells into the left flank	hUC	hUC-MSC	5, 11, 17 days after tumor cells inoculation, IV injection of 1 × 10^6^ cells or 5, 12, 19, 26, and 33 days after tumor cells inoculation, IV injection of 0.5–1 × 10^6^ cells	Reduction of adriamycin-induced side effects Improved general quality of life of animals as adjuvant therapy
Li, T. et al., 2018	Lung cancer	BALB/c nude mice; SC injection into the flank with co-cultured cells	hUC	hUC-MSC	Simultaneously with tumor cells, SC injection	Increased tumor growth by nicotine-treated hUC-MSC High heterogeneity, elevated nuclear/cytoplasmatic rations, and derangement distribution in nicotine-treated hUC-MSC tumors
Yan, C. et al., 2016	Lung cancer	BALB/c nude mice; SC injection of A549 cells into the right flank	hUC	hUC-MSC	When tumor volume was 80–120 mm^3^, IV injection of 3 × 10^5^ cells	Tumor homing capacity Higher survival rate induced by engineered hUC-MSC
Dong, L. et al., 2018	Lung cancer	BALB/c nude mice; SC injection with H1299 cells alone, H1299 cells mixed with hUCMSCs or with hUCMSC-EVs or hUCMSCs alone	hUC	hUC-MSC	Simultaneous with the injection of tumor cells, SC injection of 1.5 × 10^6^ or 6 × 10^9^ cells hUC- MSC and 200 µg of hUC-MSC-EV	Increased proliferation of cancer cells in tumor tissues Decreased apoptosis
Gauthaman, K. et al., 2012	General oncology (teratomas)	SCID mice	hUC	hUC-WJ	SC, IM, and IP Injection of 2 × 10^6^ cells/site of unlabeled human embryonic stem cells (ESC) + Matrigel, 5 × 10^6^ cells/site of fluorescence-labeled human extra-embryonic hUC-WJ or and labeled human extra-embryonic hUC-WJ + Matrigel	No tumors or inflammatory reactions induced by hUC-WJ Tumor development induced by hESCs + Matrigel Increased levels of anti-inflammatory cytokines induced by hUC-WJ
Gauthaman et al., 2013	Mammary carcinoma	SCID mice; SC injection of MDA-MB-231 cells	hUC	hUC-WJ-MSC	Protocol A: 4 days after tumor induction, IT injection of 1 × 10^6^ hUC-WJ-MSC and 100 µL of hUC-WJ-MSC -conditioned medium (50%); Protocol B: 5 weeks after tumor, IT injection of 5 × 10^6^ hUC-WJ-MSC and 100 µL of hUC-WJ-MSC-conditioned medium (50%).	Decreased tumor sizes and weights induced by hUC-WJ-MSC and hUC-WJ-MSC-CM Increased lymphocytic infiltration and vacuolation of tumor cells
Vulcano et al., 2016	Lung cancer	NOD/SCID mice	hUC	hUC-WJ-MSC	SC injection of two types of AC-LCSC or SCC-LCSC alone or co-injected with 5 × 10^6^ of hUC-WJ-MSC or Normal Human Dermal Fibroblast (NHDF)	Increased tumor size and growth induced by AC-LCSC co-inoculated with hUC-WJ-MSC Increased percentage of CD133 and CD166 Tumors with a high proliferation index No evidence of necrotic areas or pyknosis
Wu et al., 2013	Bladder cancer	BALB/c nude mice	hUC	hUC-WJ-MSC	SC injection of 1 × 10^7^ T24 cells; 1 × 10^7^ T24 cells mixed with 1 × 10^7^ of hUC-WJ-MSC; 1 × 10^7^ T24 cells mixed with 200 mg protein hUC-WJ-MSC; 200 mg protein hUC-WJ-MSC.	Decreased tumor incidence induced by hUC-WJ-MSC or MVs derived hUC-WJ-MSC co-injected with tumor cells Increased apoptosis compared to control Decreased nuclear size, extracellular matrices Decreased proliferation index
Du et al., 2014	Renal cell carcinoma	BALB/c nude mice; SC injection of 786-0 cells	hUC	hUC-WJ-MSC	Simultaneous SC injection of 1 × 10^7^ of 786-0 cells with the addition of MVs (200 µg/mL), RNase-MVs, or M199 (control).	Compared to control, increased tumor incidence and volume for animals treated with MVs derived from hUC-WJ-MSC. Enhanced expression of cyclin D1, MMP-2, and MMP-9 in tumor tissues. High proliferation index in the presence of MVs, associated with the activation of AKT and ERK1/2 signaling pathways
Mirabdollahi et al. (2020)	Breast cancer	BALB/c mice; Injection of 4T1 cells	hUC	hUC-WJ-MSC	(1) Three IV injections of hUC-WJ-MSC-derived secretome (20 mg), cisplatin (three injections, 10 mg/kg), and PBS were made for 10 days (on days 5, 15, and 15). On day 30, mice were inoculated with 3.5 × 10^6^ 4T1 cells; (2) SC injection of 3.5 × 10^6^ 4T1 cells, and when tumor appears, they received the same injections used in approach 1	Higher latency period in treatment groups Decreased tumor incidence in treatment groups Decreased tumor size and weight in secretome and cisplatin-treated groups
Chetty et al. (2020)	Melanoma	C57BL/6 mice; SC injection of B16F10 cells on the shoulder and left hind limb towards skeletal muscle	hUC	hUC-WJ-MSC	When the tumor attained the desired growth, injection of 10^6^ of hUC-WJ-MSC labeled with Mn^2+^ and Gd^3+^ co-doped CuInS2-ZnS (CIS-ZMGS) Nanocrystals (NCs)	Tumor tropism capacity No alterations in heart, kidney, or lung cells, nor liver metabolism
Vieira de Castro et al., 2017	Glioblastoma	Chicken chorioallantoic membrane assay; U251 or SNB-19 cells injected into a window made into the eggshell after puncturing the air chamber	hUC	hUC-PVC	Tumor cells were previously exposed to hUC-PVC conditioned media for 4 days, and on days 11, 13, and 15 of incubation, 100 µL of new conditioned media was added	Increased tumor growth Increased vessel density
Xie, H. et al., 2021	Hepatocellular carcinoma	BALB/c nude mice; SC injection of H1299 cells in the right flank	hUC	hUC-MSC-EV	Exosomes administered daily	Decreased tumor growth after treatment with exosomes
He, Z. et al., 2020	Esophageal cancer	BALB/c nude mice; SC injection of KYSE70 cells or EC9706 cells	hUC	hUC-MSC-EV	On days 5, 10, 15, 20, and 25, IV injection	Decreased tumor growth Increased expression of BAX and E-cadherin Decreased expression of ENAH, Bcl-2, Bcl-xl, N-cadherin, Snail stemness-related mRNAs
Jia, Y. et al., 2021	Bladder cancer	BALB/c nude mice; SC injection of cells	hUC	hUC-MSC-EV	On days 5, 10, 15, 20, and 25, an IV injection of 100 µg of hUC-MSC-EV	Decreased tumor volume and weight; decreased expression of N-cadherin, vimentin, SNAIL, Bcl-2, and PCNA; increased expression of E-cadherin and Bax
Yuan, L. et al., 2019	Breast cancer	Athymia nude mice; injection of MDA-MB- 231 cells through the mammary fat pad	hUC	hUC-MSC-EV	At days 5, 10, 15, 20, and 25, IV injection of 100 µL of hUC-MSC-EV	Decreased tumor volume Decreased expression of TRIM59, N-cadherin, vimentin, Bcl-2, BCL-xl Increased expression of Bax and E-cadherin.
Zheng, T. et al., 2022	Papillary thyroid cancer	BALB/c nude mice; SC injection of W3 cells	hUC	hUC-MSC-EV	Seven days after tumor cell inoculation, IT injection of 2 × 10^10^ hUC-MSC-EV, weekly, until day 28	Decreased tumor volume and weight after miR-30c-5p-EV treatment Decreased tumor volume and weight after injection of hUC- MSC-EV
Abello et al., 2019	Osteosarcoma	088/NUDE homozygous mice; SC injection of K7M2 cells in the lower flank	hUC	hUC-MSC	Ten days post-implantation of tumor cells, IV injection of 0.015 mmol/kg	Increased accumulation at tumor compared to Magnevist^®^
Ding et al., 2019	Pancreatic ductal adenocarcinoma	BALB/c nude mouse; SC injection of Panc-1 cells on both flanks of the mice	hUC	hUC-MSC-EV	After 7 days of tumor growth, IT injection, 3 days per week for 35 days	Decreased tumor growth Downregulation of Smad3, N-cadherin, and Bax expression; upregulation of E-cadherin, and Bcl-2 expression
Wang, Y. et al., 2021	Breast cancer	Nude mice; SC injection of MCF-7 cells	hUC	hUC-MSC-EV	10 days after tumor cell inoculation, injection of 200 µL of hUC-MSC-EV	Decreased tumor volume and weight after treatment with the inhibitor of mir-224-5p carrying exosomes
Deng, Y. et al., 2021	Pancreatic cancer	BALB/c nude mice; SC injection of Panc-1 cells	hUC	hUC-MSC-EV	Seven days after tumor growth, IT injection of 400 µL of hUC-MSC-EV every day, for three days each week	Increased tumor growth, volume, and weight with hUC-MSC-EV treatment Increased tumor growth after miR-100-5p agomir treatment
Liu, L. et al., 2021	Nasopharyngeal cancer	BALB/c nude mice; SC injection of cancer cells	hUC	hUC-MSC-EV	One week after tumor cells inoculation, SC injection	Increased tumor volume

**Legend:** SC—subcutaneous; IV—intravenous; IP—intraperitoneal; IT—intratumoral; PT—peritumoral; IM—intramuscular; GSCs—glioblastoma stem-like cells; 5-FC—5-fluorocytosine, prodrug; NMU—N-nitroso-N-methylurea; GCV—mono-phosphorylate ganciclovir, prodrug; 5-FU—5-fluorouracil; NPs—nanoparticles; HUVEC—human umbilical vein endothelial cells; SCID—severely combined immunodeficient; MSC—mesenchymal stem cells; hAF—human amniotic fluid; hAFSC—human amniotic fluid stromal/stem cells; hAF-MSC—human amniotic fluid MSC; hAM—human amniotic membrane; hAEC—human amniotic membrane epithelial cells; hAMSC—human amniotic membrane MSC; hAMTE—human amniotic membrane tissue extract; hPMSC—human placenta-derived mesenchymal stromal/stem cells; hP-ASC—human placenta-derived adherent stromal cells; hDMSC—human decidua MSC; hUC-MSC—human umbilical cord MSC; hUC-WJ-MSC—human umbilical cord Wharton’s jelly MSC; hUC-PVC—human umbilical cord perivascular cells; hUC-MSC-EV—hUC-MSC-derived extracellular vesicles.

## Data Availability

Not applicable.
